# Development and validation of the RQLQ: a Raynaud’s disease–specific measure of health-related quality of life

**DOI:** 10.1007/s10067-024-07175-0

**Published:** 2024-10-11

**Authors:** Balázs Fábián, Zoltán Csiki, Antal Bugán

**Affiliations:** 1 Department of Behavioural Sciences, Faculty of Medicine, University of Debrecen, Debrecen, Hungary; 2 Department of Internal Medicine, Faculty of Medicine, University of Debrecen, Debrecen, Hungary; 3 Clinical Psychology Center of Clinical Center, University of Debrecen, Debrecen, Hungary

**Keywords:** Health-related quality of life, Questionnaire, Raynaud’s disease, Reliability, Validity

## Abstract

**Introduction/objectives:**

The aim of this study was to develop and validate the Raynaud Specific Quality of Life Questionnaire (RQLQ) for assessing health-related quality (HRQOL) of life in patients with Raynaud’s disease (RD).

**Method:**

The questionnaire was developed and validated in three stages. Initially, semi-structured interviews with 28 RD patients identified domains of everyday life affected by RD, leading to the creation of the initial RQLQ. In the first quantitative stage, 101 patients completed the RQLQ, and exploratory factor analysis assessed dimensionality and factor structure. After removing poorly performing items, the final RQLQ was tested with 102 patients. This stage also evaluated convergent, divergent, and discriminant validity, as well as internal reliability.

**Results:**

From the interviews, 135 items were generated, with factor analysis refining the measure to 29 items across five subscales, showing good internal consistency. The RQLQ demonstrated significant correlations with self-rated quality of life and physical and mental health outcomes, confirming convergent and divergent validity. It also showed discriminant validity for different levels of disease activity.

**Conclusions:**

The RQLQ is the first specific HRQOL measure for RD patients, proving to be a psychometrically sound, reliable, and valid tool for clinical research and practice.

**Table:** 

**Key Points** *• The Raynaud Specific Quality of Life Questionnaire (RQLQ) is an important scale that evaluates the quality of life of patients with Raynaud’s disease.* *• The questionnaire showed good validity and reliability a capturing disease-specific quality of life.* *• This tool may aid in clinical research and practice.*

## Introduction

Raynaud’s disease (RD) results from episodic exaggerated vasoconstriction of arteries and arterioles in the extremities, precipitated by temperature changes and emotional distress. It usually affects the fingers and toes. Patients may experience paresthesia, hypothermia, numbness, and ischemic pain in the affected body parts. RD can be primary or secondary. RD is classified as primary when there is no underlying disease and secondary when it occurs in connective tissue diseases such as systemic lupus erythematosus, Sjögren syndrome, or systemic sclerosis [1–3]. Secondary RD often worsens progressively in extent and severity over time [4], and patients might identify persistent symptoms in between attacks of RD [5]. When the episodes are frequent and intense, they may cause obstruction of arteries, which subsequently results in irreversible structural changes that are partially or even totally resistant to healing [6].

RD is a chronic disorder that can influence patients in many ways, including their health-related quality of life (HRQOL) [7, 8]. HRQOL is broadly conceptualized as the physical, psychological, and social aspects of life that may be affected by changes in health states [9]. The patients’ perspective is of increasing importance in healthcare, and HRQOL has been included as a patient-reported outcome measure in several clinical studies [10]. Furthermore, improvement of patients’ HRQOL is mandatory for the approval of novel therapies [11].

Health-related quality of life instruments may be either generic measures of health status or disease-specific. Generic measures are designed to compare HRQOL across populations or different diseases. Examples of these generic measures include the Medical Outcomes Study 36-Item Short Form Health Survey [12] and the Euro-Qol-5D [13]. Disease-specific measures are designed to assess HRQOL with questions and scales that are specific to a disease or condition; they incorporate questions about functions typically affected by the condition [14]. Disease-specific measures quantify more clinically relevant domains than generic health status measures, as they are more sensitive to changes than generic instruments [14–16] and appropriate for evaluating specific therapeutic interventions in clinical trials [17]. There are three existing Raynaud-specific tools that were applied in previous studies: the Patient Survey of Experiences of Raynaud’s Phenomenon [5], the Raynaud Symptom Checklist [18], and the Assessment of Systemic Sclerosis–Associated Raynaud’s Phenomenon [19]. However, these scales have not been investigated for psychometric characteristics or can be only used reliably in RD when systemic sclerosis is also present.

To our knowledge, there is no standardized, comprehensive, universally accepted disease-specific HRQOL instrument available for use in patients with primary and secondary RD. This represents a serious gap, given the growing consensus about the importance of developing patient-based outcome measures [20]. Hence, the objective of the current study was to develop an acceptable and psychometrically sound HRQOL measure for patients with primary or secondary RD, using patient-centered methods.

## Material and methods

### Study design

The Raynaud-Specific Quality of Life Questionnaire (RQLQ) was developed and validated in three phases: Phase 1 was qualitative and consisted of interviews and item generation. Phases 2 and 3 were quantitative and involved the evaluation of the instrument’s psychometric properties.

In Phase 1, to identify factors reflecting patients’ health-related quality of life, the patients were asked to describe the impact of RD and its treatment on their daily life, including physical functioning, emotional well-being, social interactions, and work. Interviews continued until no new issues emerged. Transcripts were analyzed qualitatively to identify participants’ expressions of domains. The authors independently proposed domains after reviewing the transcripts. Afterward, differences in domains were discussed until a final agreement was reached. Based on the domains obtained, items were phrased by the authors and used as the first form of the questionnaire. The process of item development was the same as the domain development. Each item was carefully worded to ensure that it related specifically to RD, and where possible, the patients’ terminology was used. The initial set of items was reviewed by some of the patients and medical professionals experienced in working with patients with RD. Based on their comments, items were reviewed and, if necessary, reworded. The questionnaire instructions and a 5-point Likert response format were devised.

We followed a generally accepted approach [21] for the development and validation of health measurement scales. The conduct of this study adhered to the principles outlined in the Declaration of Helsinki. All data were obtained anonymously. Informed written consent was obtained from all patients before study enrollment. This study received approval from the Regional and Institutional Ethics Committee and the Scientific and Research Ethics Committee (registration number: RKEB/IKEB 4822–2017).

### Participants

The study was conducted among consecutive patients with RD at Raynaud’s Outpatient Clinic of the Department of Internal Medicine. The inclusion criteria and the diagnostic criteria of RP were the same in every phase of the study. Inclusion criteria included being able to read and write Hungarian and being willing to participate in the study. We excluded patients if they received psychiatric medication, participated in regular psychiatric care, or had any difficulties with consciousness, language, or cognition that would prevent reliable completion of the questionnaire. RD was diagnosed based on the criteria established by Maverakis et al. [22]. We applied nailfold capillaroscopy and anti-nuclear antibody (ANA) testing, took detailed medical history, and made observations of blanching and/or cyanosis [23, 24]. To prevent any bias related to outside temperature and weather, data collection for all three phases was conducted under similar weather conditions during the fall and spring seasons, when outside temperature is neither too cold nor too warm. Additionally, all study participants were assessed at a single center in the same country with homogeneous weather conditions.

### Measures

#### Brief beck depression inventory

The Beck Depression Inventory (BDI) is a self-report inventory that measures symptoms of depression. In the present study, the Hungarian brief version was used, which is nine items long and demonstrated sound psychometric properties in both nonclinical and clinical samples [25].

#### WHO-five well-being index

The WHO-Five Well-Being Index (WBI) is a short, positively worded instrument consisting of 5 simple and non-invasive questions designed to assess the subjective psychological well-being of the respondents [26].

#### Generalized anxiety disorder scale

The 7-item Generalized Anxiety Disorder Scale (GAD-7) is a one-dimensional scale designed to assess the presence of the symptoms of Generalized Anxiety Disorder, as listed in the DSM-IV [27].

#### Perceived stress scale

The Perceived Stress Scale (PSS) is a 10-item self-report measure that assesses the amount of perceived psychological stress over the previous month [28].

#### Pain severity

Pain was assessed using three questions from the Hungarian version of the SF-36 questionnaire. These items were modified for RD, similar to a previous study [29]. Two of the three questions were answered on a 4-point scale, and the third item was answered on a 5-point scale. The final raw scores (ranging from 3 to 13) were rescaled to range from 5 to 100 according to the Manual of SF-36. A higher score indicated a higher degree of pain.

#### Symptom severity

To assess symptom severity, the following question was used: “How do you perceive the severity of your Raynaud’s Disease?”. The item was scored on a 5‐point Likert rating format from 1 (not serious at all) to 5 (serious disease severity).

### Statistical analysis

Statistical analyses were performed using SPSS v. 22 (SPSS Inc., IBM, Chicago, IL, USA) and R version 3.2.3 software. The normality of the data was checked by the Kolmogorov–Smirnov test. The rate of missing data was below 0.1%. Missing data were replaced by the respective sample mean for continuous variables. Associations with validity measures were assessed using Pearson’s correlation coefficients or Spearman’s rank order correlation coefficients, as appropriate.

Factor analysis (principal component extraction and promax rotation) of the initial items was used to identify the most appropriate items. In Phase 2, the generation of factors was exploratory (EFA), so no restriction was placed on the number extracted. Internal reliability was assessed using Cronbach’s alpha coefficients. Items were systematically removed one at a time, in accordance with the following criteria: items with high loadings on more than one factor (factor loadings over 0.4 on at least two scales) and items demonstrating factor loading below 0.6 were eliminated. After removing items from the item pool, EFA was repeated until every item met the criteria presented above. Additionally, each identified domain was examined to see if removal of any item led to an increase in its internal consistency.

In Phase 3, the results of confirmatory factory analysis (CFA), principal component analysis (PCA), scree plots, and eigenvalues were used to assess optimal simple structure of the final form of the scale. Criteria for good model fit were determined by comparative fit index (CFI) > 0.9, incremental fit index (IFI) > 0.9, Tucker-Lewis Index (TLI) > 0.9, Root Mean Squared Error of Approximation (RMSEA) < 0.07, and standardized root means residual (SRMR) < 0.08. We also report normal theory χ2 though with large samples these figures are likely to be significant at *p* < 0.05 even when other fit indices are suggesting reasonable fit. Relative chi-square (chi-square/df) was used as a correction for the chi-square test, with a cutoff score of below 3.00 [30–32].

For the final version of RQLQ and its subscale mean scores, standard deviations, skewness, kurtosis, and percentages of respondents scoring the minimum (floor) and maximum (ceiling) possible scores were calculated to evaluate scale score distributions. Floor effects were deemed present if more than 20% of patients endorsed the lowest possible score, and ceiling effects were deemed present if more than 20% of patients endorsed the highest possible score.

The convergent and divergent validity of the RQLQ was assessed by examining the association of RQLQ scores with well-established questionnaires of physical and mental health outcome measures*.* We predicted that those with a worse RD-related quality of life would have higher levels of depression, anxiety, perceived stress, RD-related pain, and lower well-being.

To examine the discriminant (known groups) validity, the RQLQ and subscale scores were compared in different groups of patients according to their perceived symptom severity. It was predicted that patients with higher degrees of self-reported disease severity in winter would have worse RQLQ scores since cold is one of the two main triggering factors of RD [1]. We used Kruskal–Wallis test to examine the differences across these groups.

## Results

### Demographic and clinical characteristics of participants

In Phase 1, 64.39% of the participants were women. The mean age was 53.5, with a range of 19–70. 46.43% of participants were diagnosed with secondary RD with single or multiple RD-related conditions. A total of 101 respondents with RD took part in Phase 2 and a total of 102 respondents with RD (12 male and 90 female) took part in Phase 3 (see Table 1).

**Table 1:** Demographic and clinical characteristics of participants in Phases 2 and 3

	Phase 2 (*N* = 101)	Phase 3 (*N* = 102)
Mean ± SD (range); number (%)		
Age, year	50.5 ± 15.3 (19–82)	53.68 ± 13.5 (18–82)
Gender, women	87 (86.14)	90 (88.2)
Education, year	12.53 ± 2.56	12.86 ± 2.74
Family status, single	26 (25.74)	29 (28.4)
Employment status, employed	53 (52.5)	42 (41.2)
Clinical data		
RD type		
Primary RD	60 (59.01)	46 (45.1)
Secondary RD	41 (40.59)	56 (54.9)
Primary disease (only in secondary RD)		
Rheumatoid arthritis	33 (80.5)	27 (37.5)
Carpal tunnel syndrome	10 (24.4)	14 (25)
Sjögren syndrome	11 (26.8)	25 (44.6)
Crohn disease	1 (2.4)	0 (0)
Systemic lupus erythematosus	0 (0)	2 (3.6)
Systemic sclerosis	1 (2.4)	5 (8.9)

*SD*, standard deviation

### Phase 1: questionnaire construction

The thematic analysis of the transcripts resulted in the following main themes: experiences of the symptoms, decreased functionality, treatment and prevention, relationships, emotional and cognitive difficulties, work, travel, and sleep. For each domain, items were generated based on the interview transcripts. The item generation process resulted in 135 initial items.

### Phase 2: questionnaire validation

#### Construct validity

In Phase 2, EFA and internal reliability assessment were used repeatedly for item selection, as described in the previous section, resulting in a selection of 29 items. For the final EFA, the Kaiser–Meyer–Olkin value was 0.927, exceeding the recommended value of 0.6, and Bartlett’s Test of Sphericity was statistically significant (χ2 = 3039.72; *p* < 0.001), supporting the factorability of the final 29-item correlation matrix. The five-factor structure accounted for 76% of the overall variance within the data set. The number of domains determined in the first phase decreased from eight to five. The domains identified were Impaired Hand Function (8 items), Social Interaction (8 items), Emotional Burden (6 items), Control (4 items), and Sleep (3 items). Internal reliability is perceived as good for all domains, ranging between 0.88 and 0.97. Also, all factor correlations were significant, ranging between 0.417 and 0.632 (see Table 2).

**Table 2:** Scale correlations and descriptive statistics for Phase 2

Scale	1	2	3	4	5	6
1. RQLQ	(0.97)					
2. RQLQ-Impaired Hand Function	0.85*	(0.96)				
3. RQLQ-Social Interaction	0.82*	0.52*	(0.95)			
4. RQLQ-Emotional Burden	0.83*	0.59*	0.65*	(0.95)		
5. RQLQ-Control	0.64*	0.43*	0.44*	0.41*	(0.88)	
6. RQLQ-Sleep	0.80*	0.71*	0.49*	0.57*	0.53*	(0.92)

Numbers on the diagonal are Cronbach’s alpha. *RQLQ*, Raynaud Specific Quality of Life Questionnaire. *p < 0.005

In Phase 3, to confirm the factor structure of the RQLQ’s subscales, CFA was performed. First, in order to check sample adequacy, the Kaiser–Meyer–Olkin Measure of Sampling Adequacy (KMO) index and Bartlett’s Test of Sphericity (BTS) were conducted before the factor analysis. Both the overall KMO for the scale (0.91) and BTS (χ^2^ = 3002.86; *p* < 0.001) suggested the data was appropriate for factor analysis.

The CFA indicated that the five-factor solution provided an acceptable fit. All items met the criteria used in Phase 2 for item selection. An examination of the fit indices indicated that the five-factor solution provided an acceptable fit. The model fit indices for this model were as follows: CFI = 0.90; IFI = 0.90; TLI = 0.9; RMSEA = 0.09; SRMR = 0.065. This indicates good global model fit, although it should be noted that RMSEA has a little difference from the rule of thumb cut-off point used for assessing acceptable model fit. All items had a robust loading on their respective factor, ranging from 0.62 to 0.95 (see Table 3), and factor correlations were significant, ranging between 0.370 and 0.671, suggesting an acceptable measurement model for the five-factor model. The five-factor solution explained 74% of the variance. The Chi^2^ test was also calculated but, due to its statistic’s sensitivity to sample size, relative chi-square (CMIN) was calculated (Chi^2^/df) as a correction for the chi-square test. CMIN was 1.83 indicating good fit. According to the scree plot, the break above the elbow occurred after the first factor. Additionally, the PCA indicated the five-factor solution was appropriate since the first five eigenvalues were greater than 1 (14.84, 2.54, 2.39, 1.69, and 1.23). Based on these results, we decided to keep all the 29 items retained in Phase 2.

**Table 3:** Factor loadings for the 5 domains of the RQLQ for Phase 3

	Factor				
Item	1	2	3	4	5
3. Due to my hand complaints, I am progressing more slowly with my work. (3. A kéz panaszaim miatt lassabban haladok a munkámmal.)	**0.95**	− 0.04	− 0.03	0.00	− 0.06
23. My illness hinders my grip on work tools. (23. A munkaeszközök fogásában hátráltat a betegségem.)	**0.93**	− 0.10	0.04	0.01	− 0.02
4. Due to the stiffness of my hands, I have difficulty gripping properly. (4. A kezeim merevsége miatt nehezen tudok rendesen fogni.)	**0.89**	− 0.02	0.11	0.02	− 0.06
2. Fine, small movements are difficult for me. (2. A finom, apró mozdulatok nem mennek.)	**0.88**	− 0.00	− 0.03	− 0.05	0.00
1. My illness limits my grip strength. (1. A betegségem korlátoz a szorításban.)	**0.87**	0.07	− 0.11	− 0.06	0.03
20. I have difficulty holding smaller objects. (20. Nehezen fogok meg apróbb tárgyakat.)	**0.83**	0.15	− 0.01	0.01	− 0.02
14. I can grip with less strength. (14. Gyengébben tudok fogni.)	**0.71**	0.01	0.10	− 0.01	0.13
19. It’s hard to grip anything. (19. Nehéz bármit is megfogni.)	**0.70**	0.17	− 0.04	0.04	0.12
9. I am ashamed of my illness in front of others because of the color of some parts of my body. (9. Egyes testrészeim színe miatt szégyellem a többi ember előtt a betegségem.)	− 0.03	**0.91**	− 0.01	− 0.05	0.02
26. I am ashamed in front of others of how the affected area (e.g., my hands) looks due to my illness. (26. Szégyellem mások előtt azt, ahogy a betegség által érintett terület (pl. a kezeim) kinéz.)	− 0.01	**0.89**	0.00	0.10	− 0.11
25. I am ashamed of the condition of some parts of my body because I look like I am old. (25. Szégyellem egyes testrészeim állapotát, mert úgy nézek ki, mintha öreg lennék.)	0.08	**0.89**	− 0.03	0.01	− 0.08
17. I would prefer to always hide my hands so that others never see them. (17. Legszívesebben mindig eldugnám kezeimet, hogy mások sose lássák.)	− 0.03	**0.82**	− 0.05	0.04	− 0.02
8. I have to hide my hands because I don’t want to know how others would react. (8. Dugdosnom kell a kezeimet, mert nem akarom tudni, hogy reagálna rá a többi ember.)	0.02	**0.78**	0.11	0.02	− 0.05
24. I have to conceal the body parts affected by my illness. (24. Rejtegetnem kell a betegségem által érintett testrészeimet.)	0.12	**0.78**	− 0.19	− 0.03	0.05
16. It annoys me that I have to hide some parts of my body, like my hands. (16. Idegesít, hogy dugdosnom kell egyes testrészeimet, pl. a kezeimet.)	− 0.03	**0.73**	0.20	0.01	0.01
29. It bothers me how some parts of my body look because I find them ugly. (29. Zavar, hogy ahogy egyes testrészeim kinéznek, mert csúnyának tartom őket.)	− 0.00	**0.71**	− 0.00	− 0.01	0.17
12. I am afraid of what will happen if my symptoms worsen. (12. Félek attól, hogy mi lesz, hogyha a tüneteim súlyosbodni fognak.)	− 0.12	0.07	**0.95**	− 0.06	− 0.03
6. I worry if my complaints will limit me even more later. (6. Aggódok, hogy később még jobban korlátozni fognak-e a panaszaim.)	0.15	− 0.19	**0.92**	0.10	− 0.05
5. I am concerned that my condition will worsen. (5. Aggódok, hogy állapotom rosszabbodni fog.)	0.03	− 0.13	**0.91**	0.06	0.02
13. It upsets me that I can’t defend against my complaints. (13. Felzaklat, hogy nem tudok védekezni a panaszaimmal szemben.)	− 0.08	0.16	**0.72**	− 0.00	0.14
10. It saddens me that I am sick. (10. Elkeserít, hogy beteg vagyok.)	0.08	0.27	**0.64**	− 0.01	− 0.15
11. It makes me sad that I can’t always do something to alleviate my complaints. (11. Elszomorít, hogy nem minden esetben tudok tenni valamit, hogy panaszaim enyhüljenek.)	− 0.01	0.22	**0.64**	− 0.14	0.13
28. I wear thicker clothes. (28. Vastagabb ruhákban járok.)	0.03	− 0.03	− 0.06	**0.93**	0.02
27. I have to wear more or thicker socks. (27. Több vagy vastagabb zoknit kell felvennem.)	− 0.18	0.09	− 0.05	**0.80**	0.15
18. I always have to warm up the place (e.g., room or car) where I stay. (18. Mindig fel kell melegítenem azt a helyet (pl. szoba vagy autó) ahol tartózkodok.)	0.01	0.01	0.02	**0.72**	− 0.07
21. At home, I hide under the blanket whenever possible. (21. Otthon a takaró alá bújok, amikor csak lehet.)	0.11	0.01	0.11	**0.70**	− 0.07
15. I wake up at dawn with cold or numb hands. (15. Hajnalban arra ébredek, hogy hidegek vagy zsibbadnak a kezeim.)	− 0.03	0.01	0.00	0.03	**0.94**
7. During the night, I wake up due to numbness. (7. Az éjszaka folyamán zsibbadásra ébredek.)	0.05	− 0.06	− 0.01	− 0.03	**0.92**
22. While resting, my hands feel numb or painful. (22. Pihenés közben zsibbadnak vagy fájnak a kezeim.)	0.27	0.02	0.05	0.06	**0.62**

Pattern coefficients greater than 0.4 are shown in bold to facilitate interpretation. *RQLQ*, Raynaud Specific Quality of Life Questionnaire

Based on this sample, only one domain (Social Interaction) may have a significant potential for a ceiling effect, but there were no significant floor effects. There were 1 skewness (− 1.12) and 3 kurtosis (between − 1.32 and − 1.08) statistics that fell outside the criteria of ± 1, representing “very good” symmetry of a normal univariate distribution (see Table 4).

**Table 4:** Descriptive statistics for Phase 3

Scale	No. of items	Mean	SD	Skewness	Kurtosis	% floor	% ceiling
RQLQ	29	92.54	27.96	− 0.31	− 0.79	0.9	0.9
RQLQ-Impaired Hand Function	8	23.82	9.93	0.02	− 1.28	5.5	3.6
RQLQ-Social Interaction	8	32.01	9.18	− 1.12	0.37	3.6	29.1
RQLQ-Emotional Burden	6	16.86	6.67	0.19	− 0.90	5.5	1,8
RQLQ-Control	4	10.89	4.78	0.25	− 1.08	9.1	4.5
RQLQ-Sleep	3	8.97	4.13	0.06	− 1.32	12.7	13.6

*RQLQ*, Raynaud Specific Quality of Life Questionnaire; *SD*, standard deviation

### Convergent and divergent validity

The RQLQ scale and its subscales showed good validity with the applied outcome measures of emotional and physical well-being: all correlations were moderate, and they were in the predicted direction and achieved statistical significance (see Table 5). The RQLQ and all five domains demonstrated a moderate negative association with self-rated depression, anxiety, perceived stress, and pain, and a moderate positive association with general well-being (Table 5). The strongest correlate of BDI (*r* =  − 0.59), GAD-7 (*r* =  − 0.39), and PSS (*r* =  − 0.50) was the RQLQ total score. WBI showed the strongest relationship with the Emotional Burden subscale (*r* =  − 0.53), while the strongest correlate of pain severity was the Impaired Hand Function subscale (*r* =  − 0.72). Every subscale of RQLQ showed the strongest association with the measure of pain, except for the Control subscale whose correlation was the strongest with BDI (*r* =  − 0.36).

**Table 5:** Scale correlations for Phase 3

Scale	1	2	3	4	5	6	7	8	9	10	11
1. RQLQ	(0.96)										
2. RQLQ-Impaired Hand Function	0.87**	(0.96)									
3. RQLQ-Social Interaction	0.87**	0.64**	(0.95)								
4. RQLQ-Emotional Burden	0.82**	0.62**	0.69**	(0.94)							
5. RQLQ-Control	0.57**	0.36**	0.40**	0.35**	(0.87)						
6. RQLQ-Sleep	0.76**	0.66**	0.54**	0.52**	0.43**	(0.92)					
7. BDI	− 0.59**	− 0.51**	− 0.47**	− 0.54**	− 0.36**	− 0.44**	(0.90)				
8. WBI	0.49**	0.36**	0.43**	0.53**	0.24**	0.32**	− 0.50**	(0.88)			
9. GAD-7	− 0.39**	− 0.33**	− 0.32**	− 0.36**	− 0.24*	− 0.29**	0.54**	− 0.41**	(0.93)		
10. PSS	− 0.50**	− 0.43**	− 0.39**	− 0.48**	− 0.30**	− 0.34**	0.54**	− 0.25*	0.49**	(0.80)	
11. Pain	0.69**	0.72**	0.49**	0.57**	0.35**	0.61**	− 0.58**	0.53**	− 0.39**	− 0.28*	(0.92)

Numbers on the diagonal are Cronbach’s alpha. *RQLQ*, Raynaud Specific Quality of Life Questionnaire; *BDI*, Brief Beck Depression Inventory; *WBI*, WHO-Five Well-Being Index; *GAD-7*, Generalized Anxiety Disorder Scale; *PSS*, Perceived Stress Scale. *p < 0.01. **p < 0.005

#### Discriminant (known groups) validity

To assess discriminant validity analysis, the RQLQ and subscale scores were compared in three different groups of patients according to their symptom severity: group 1 included patients with very serious disease severity (*N* = 13), group 2 included patients with serious disease severity (*N* = 36), and group 3 included patients with moderate disease severity (*N* = 36). Patients reporting better than moderate health status in connection with RD symptoms (*N* = 2), or not answering this question (*N* = 15), were excluded from the discriminant validity analysis. As can be seen in Table 6, significant differences were found between groups. In all cases, the mean RQLQ scores were in the expected direction: those with a higher self-rated disease severity had a lower RQLQ total score (*p* < 0.001), as well as lower RQLQ sub-scale scores (*p* < 0.001).

**Table 6:** Comparsions of the RQLQ and its subscales by self-perceived symptom severity

	Symptom severity				
	Very serious (*n* = 13)	Serious (*n* = 36)	Medium (*n* = 36)	χ^2^	*p*-value
	Mdn (IQR)	Mdn (IQR)	Mdn (IQR)		
RQLQ	63 (31.82)	83.72 (35.72)	116.5 (29.42)	33.67	< 0.001
RQLQ-Impaired Hand Function	12 (7.5)	17.59 (16.65)	31 (11.75)	28.86	< 0.001
RQLQ-Social Interaction	26 (19.09)	31.2 (17.37)	40 (4.5)	23.87	< 0.001
RQLQ-Emotional Burden	12 (10.64)	13 (7.82)	21 (8.12)	20.55	< 0.001
RQLQ-Control	6 (3)	10 (4.95)	14 (8.75)	19.42	< 0.001
RQLQ-Sleep	6 (3.6)	8 (6)	12 (7.36)	18.34	< 0.001

*Mdn*, median; *IQR*, interquartile range; *RQLQ*, Raynaud Specific Quality of Life Questionnaire

## Discussion

Generic instruments are designed to assess HRQOL across a wide variety of medical conditions. The disease specificity of the measure may provide important additional information regarding the patient’s subjective health status and can help detect significant changes in quality of life. While patients with primary RD can develop connective tissue diseases [33], repeated assessment of quality of life might help to initiate preventative treatments early and modify risk factors. In secondary RD, measuring changes in quality of life indicates disease progression and treatment effectiveness. Since no Raynaud’s disease-specific HRQOL measures existed, in this study, we developed a disease-specific HRQOL instrument for patients with primary and secondary RD using qualitative and quantitative methods.

In the first quantitative phase, the number of items was reduced from 135 to 29 items. The results from factor analyses revealed that the five-factor solution is appropriate, and ceiling and floor effects were also shown to be acceptable. We considered the five-factor structure clinically relevant and conceptually acceptable. The final five factors of the RQLQ were Impaired Hand Function, Social Interaction, Emotional Burden, Control, and Sleep. These domains identified are consistent with previous studies exploring patient experiences in RD [20, 34, 35]. For further research, we recommend researchers computing average scores if they want to make comparisons between subscales, and we also recommend using both subscale and total scores. Indices of internal consistency reliability for the RQLQ total score and all RQLQ domains at both phases were well above recommended standards (i.e. > 0.70), suggesting a good construct validity.

Convergent and divergent validity were supported by the finding that significant, but moderate correlations with measures of well-being, depression, anxiety, stress, and pain were always found in the expected direction. These results are in line with previous findings [8], which reported significant associations between quality of life and measures of stress, anxiety, depression, and symptom severity. Based on these results, we argue that referral to rehabilitation services and/or psychological care might help the patients to manage mental health and emotional issues and to learn adaptive behavioral strategies in symptom management. However, developing and testing efficient behavior change interventions is warranted [36].

Evidence of discriminant validity was provided by the significant differences in RQLQ scores depending on perceived disease severity. These results further confirm other studies’ findings, which reported that using a disease-specific tool to measure HRQOL is practically useful and represents an important line of research. For example, similar achievements have been obtained by developing disease-specific tools in other studies involving patients with chronic diseases such as Sjögren’s syndrome [37], rheumatoid arthritis [38], upper limb lymphedema [39], and psoriatic arthritis [40].

There are some limitations to the study. The first limitation of the study was the small number of participants in Phase 2 (*N* = 102) compared to the initial number of items (135). However, in Phase 3, the number of participants (*N* = 101) was sufficient for CFA with a 29-item measure to establish a final structure. However, we recommend conducting further studies using confirmatory factor analysis with larger sample sizes to validate our results. The second limitation of the psychometric testing of the questionnaire is the relatively small number of male participants. Our samples were predominantly female (approximately 90%), which may have influenced scale development. However, a higher percentage of men were involved in the first phases of the questionnaire development. In Phase 1, 35.7% of the 28 participants were men (*n* = 10), and their comments were given due consideration during item generation and revision. Furthermore, during Phases 2 and 3, all approached patients agreed to participate in the study except for two men and three women. Therefore, the results are highly representative of the patient population visiting the Raynaud’s Disease Outpatient Clinic of the Department of Internal Medicine. These observations are in contrast with the previously reported epidemiological data. According to previous studies, the prevalence of RD in the general population is 3–6% among men and 4–9% among women [41, 42]. The main reason for this difference in our study could be that women are more likely to show help-seeking behavior than men [43]. Additionally, the test–retest reliability of the final 29-item version of the RQLQ was not assessed in the current study. Further investigations are needed to assess the responsiveness (i.e., the ability to detect even small but important changes over time) of the RQLQ. Future longitudinal research could improve the validity of the RQLQ, as primary RD can evolve into secondary RD [44], and secondary RD may worsen in severity over time [45]. In our samples, patients were diagnosed with various, sometimes multiple RD-related disorders with different treatments. This might be also considered as a limitation; however, RQLQ is designed to assess disease-specific quality of life regardless of the primary disease or treatment, and we did not conduct any subgroup comparisons or examine treatment effects. The effect of cold weather on the RQLQ should be also investigated by comparing data collected during the summer and winter months.

In conclusion, the RQLQ is the first valid and reliable disease-specific HRQOL instrument for patients with primary and secondary RD. Although some further confirmation of the factor structure and the evaluation of the test–retest reliability is recommended, the RQLQ may serve as a clinically meaningful outcome measure in research and clinical practice.

## Data Availability

The data that support the findings of this study are available from the corresponding author, B. F., upon reasonable request.
